# Recent Advances in g-C_3_N_4_-Based Materials and Their Application in Energy and Environmental Sustainability

**DOI:** 10.3390/molecules28010432

**Published:** 2023-01-03

**Authors:** Qian Wang, Yongfei Li, Fenglin Huang, Shaofu Song, Ganggang Ai, Xin Xin, Bin Zhao, Yajun Zheng, Zhiping Zhang

**Affiliations:** 1 School of Chemistry and Chemical Engineering, Xi’an Shiyou University, Xi’an 710065, China; 2 Xi’an Key Laboratory of Low-Carbon Utilization for High-Carbon Resources, Xi’an Shiyou University, Xi’an 710065, China; 3 State Key Laboratory of Petroleum Pollution Control, Xi’an Shiyou University, Xi’an 710065, China; 4 National Center for Quality Supervision and Inspection of Oil and Gas Products (Yan’an), Yan’an 716000, China; 5 Department of Crop Soil Sciences, Washington State University, Pullman, WA 99164-6420, USA; 6 Department of Statistics, North Dakota State University, Fargo, ND 58105, USA

**Keywords:** g-C_3_N_4_, preparation, modification, hydrogen evolution, CO_2_ conversion, organic pollutants

## Abstract

Graphitic carbon nitride (g-C_3_N_4_), with facile synthesis, unique structure, high stability, and low cost, has been the hotspot in the field of photocatalysis. However, the photocatalytic performance of g-C_3_N_4_ is still unsatisfactory due to insufficient capture of visible light, low surface area, poor electronic conductivity, and fast recombination of photogenerated electron-hole pairs. Thus, different modification strategies have been developed to improve its performance. In this review, the properties and preparation methods of g-C_3_N_4_ are systematically introduced, and various modification approaches, including morphology control, elemental doping, heterojunction construction, and modification with nanomaterials, are discussed. Moreover, photocatalytic applications in energy and environmental sustainability are summarized, such as hydrogen generation, CO_2_ reduction, and degradation of contaminants in recent years. Finally, concluding remarks and perspectives on the challenges, and suggestions for exploiting g-C_3_N_4_-based photocatalysts are presented. This review will deepen the understanding of the state of the art of g-C_3_N_4_, including the fabrication, modification, and application in energy and environmental sustainability.

## 1. Introduction

Along with the rapid growth of the global population and the development of industrialization and urbanization, the demand for fossil energy, such as petroleum, coal, and natural gas, is increasing, as well as the deterioration of environmental pollution [1]. Driven by the ongoing energy and ecological crisis, the development of sustainable energy is a matter of great urgency and is related to the vital interests of people worldwide. As a green and renewable energy, solar energy has become a hot topic [2] and converting it into chemical energy by using photocatalysis is regarded as a potential pathway to supply renewable energy and alleviate environmental issues in the future. The photocatalyst is the most important key in economic photocatalysis application; it should be efficient, stable, low-cost, and capable of harvesting visible light [3]. Many photocatalytic materials have been reported and used in various fields, including hydrogen evolution, contaminant photo-oxidization or photodecomposition, and photoelectrochemical conversion [4,5]. For example, titanium dioxide (TiO_2_) and related photocatalysts have been used in solar energy conversion, due to their merits of low price, unique optical-electronic properties, great durability, and non-toxicity [6]. However, the large bandgap of TiO_2_ (3.2 eV) prevents it from actual solar energy utilization [7,8]. Compared to TiO_2_, the newly emerged graphitic carbon nitride (g-C_3_N_4_) is a visible-light-response material with a narrower bandgap of 2.7 eV. It is a kind of metal-free photocatalyst and possesses the advantages of simple synthesis, suitable semi-conducting properties, and high structural stability under both thermal and photochemical conditions. These merits make g-C_3_N_4_ a unique material for energy and environmental applications, including photocatalytic H_2_ generation, CO_2_ reduction, and degradation of organic pollutants (dyes, pesticides, pharmaceuticals, phenolic compounds) and inorganic pollutants like heavy metals, carbon dioxide reduction, etc. [6,9,10,11,12,13,14]. In 2009, Wang et al. [15] for the first time proved that g-C_3_N_4_ could be used for photocatalytic hydrogen production upon visible-light irradiation, marking a significant milestone in metal-free photocatalysts. Nevertheless, due to the rapid recombination of photo-induced charges, the photocatalytic performance of g-C_3_N_4_ still possesses a significant possibility to be further enhanced, and the research on g-C_3_N_4_ gives rise to a new upsurge. So far, different strategies have been adopted to increase the photocatalytic efficiency of g-C_3_N_4_, such as morphological control, element doping, heterojunction construction, and nanomaterial composition [8,16,17]. In this review, we introduce the recent advances in the preparation and modification of g-C_3_N_4_, and summarize its photocatalytic application in H_2_ generation, CO_2_ reduction, and degradation of organic pollutants in recent years. Finally, we conclude the research challenges with g-C_3_N_4_ and suggest perspectives for future research direction.

## 2. Properties and Preparation of g-C_3_N_4_

### 2.1. The Origin and Properties of g-C_3_N_4_

**Figure 1:**
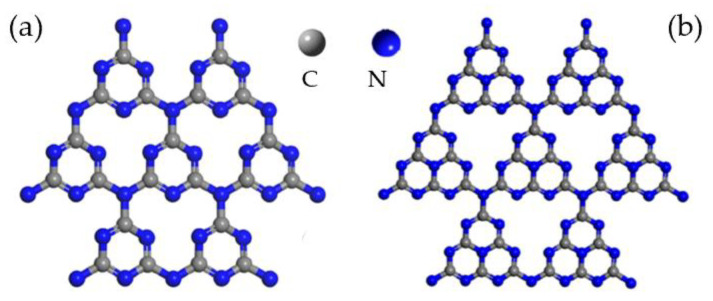
Structures of (**a**) triazine and (**b**) tri-s-triazine. Reprinted with permission from Ref. [25]. Copyright 2021 American Chemical Society.

Carbon nitride (C_3_N_4_), composed of carbon and nitrogen elements, is a kind of organic semiconductor material. The history of C_3_N_4_ can be traced back to 1834. One type of material named “melon” was synthesized by Berzelius and also reported by Liebig, which is a linear polymer connected triazine and tri-s-triazines (Figure 1) via secondary nitrogen [18,19]. However, this material did not attract much attention due to the lack of comprehensive characterization at the time. Along with the development of characterization methods, Franklin [20] probed this material in 1922 and proposed the concept of C_3_N_4_, which indicated that C_3_N_4_ could be obtained by polymerizing various ammonia carbonic acids. Later, Pauling and Sturdivant [21] demonstrated that tri-s-triazine was the unit of melon in 1937. Still, its chemical instability and insolubility in most reagents made it impossible to unveil the structure until 1989, researchers found that when Si in β-Si_3_N_4_ was replaced by C, the derived β-C_3_N_4_ was as hard as a diamond [22]. Based on the study, five types of C_3_N_4_, comprising α, β, pseudocubic, cubic, and graphitic, were predicated in 1996 [23]. Among them, the first four materials are hard materials but not favorable to be synthesized due to their low stability [24] and g-C_3_N_4_ is confirmed as the most resistant under surrounding situations; it possesses a similar layered structure to graphene and sp2 hybrid π-conjugated electronic band structure. In recent years, large numbers of g-C_3_N_4_ materials have been synthesized by thermal polymerization of urea, melamine, cyanamide, dicyandiamide, and thiourea, which indicated that g-C_3_N_4_ was composed of melem units and further confirmed that tri-s-triazine was the basic unit of g-C_3_N_4_.

The electronic structures of both carbon and nitrogen atoms in g-C_3_N_4_ determine their electronic and optical properties. The lone pair electrons of carbon and nitrogen atoms of g-C_3_N_4_ interact to create a large π bond, analogous to the benzene ring, and then form a highly delocalized conjugate system. The delocalized conjugate chemical structure contributes to the formation of the stacking of the carbon nitride layer, which connects through amines, and such a structure makes the superior electronic conductivity of g-C_3_N_4_ [1]. In addition, the solid covalent bond between carbon and nitrogen atoms leads to the excellent chemical and thermal stability of g-C_3_N_4_. After experimental measurements [26,27] the conduction band (CB) and valence band (VB) of g-C_3_N_4_ are −1.3 V and 1.4 V at pH = 7 versus the standard hydrogen electrode (SHE), respectively. Such band positions promote visible light harvesting under oxidation and reduction systems. In short, g-C_3_N_4_ possesses unique electronic, structural, physicochemical, and optical properties, sufficient for photocatalytic application in H_2_ production, CO_2_ photoreduction, and degradation of organic pollutants [9,28].

### 2.2. Preparation of g-C_3_N_4_

The property of a catalyst varies substantially depending upon the preparation protocols. To achieve the application of g-C_3_N_4_ in the field of photocatalysis, the synthesis of high-performance g-C_3_N_4_ is a prerequisite. Various methods have been proposed, including thermal condensation, hydrothermal and solvothermal approaches, solid-state fabrication, self-hand synthesis, template-supported formation, deposition-precipitation, and ball milling process [1,29].

Among these methods, thermal condensation, a combination of polycondensation and polyaddition, is the most common method to prepare g-C_3_N_4_. Nitrogen-rich chemicals, such as melamine, urea, and dicyandiamide, are usually used as precursors, and after the deamination process, g-C_3_N_4_ is generated under high temperatures. For example, Yan et al. [30] synthesized g-C_3_N_4_ with a high photodegradation activity toward methyl orange in a semiclosed system with a two-step heat treatment. By investigating the influence of heating temperature upon the thermal condensation of melamine, the optimal reaction condition for g-C_3_N_4_ was 520 °C for 2 h. Liu et al. [31] used urea as a precursor and produced g-C_3_N_4_ on a large scale by pyrolysis under ambient pressure without additive assistance. The retainable pyrolysis-generated self-supporting atmosphere and the reaction temperature are two necessary conditions.

Hydrothermal synthesis is also one of the most widely used methods, which is beneficial to control the accuracy in influencing reaction molar ratio and physio-chemical properties [1]. Wu et al. [32] prepared oxygen-containing-groups-modified g-C_3_N_4_ (OG/g-C_3_N_4_) through an in situ one-step hydrothermal treatment of bulk g-C_3_N_4_ in pure water. Hydrothermal treatment at 180 °C could promote the increase in the specific surface area of the resulting product from 2.3 to 69.8 m^2^ g^−1^, and oxygen-containing groups (-OH and C=O) were also successfully grafted on the surface of OG/g-C_3_N_4_ via the interlayer delamination and intralayer depolymerization. Due to its high surface area and oxygen-containing surface properties, OG/g-C_3_N_4_ demonstrated high photocatalytic performance on H_2_ evolution. Ahmad et al. [33] synthesized a highly efficient double *Z*-scheme g-C_3_N_4_/AgI/β-AgVO_3_ (g-CNAB) ternary nanocomposite using a one-pot hydrothermal route. They have characterized the optical properties, phase structure, and morphology of the as-prepared photocatalysts and evaluated their photocatalytic performance toward the photodegradation of different pollutants under visible-light irradiation. Experimental characterization indicated that g-CNAB possessed a dual *Z*-scheme heterojunction, which had the features of better spatial separation and charge-carrier transfer. As such, reactive species such as superoxide anion radical and hydroxyl radical can be favorably generated for the degradation of various contaminants.

**Figure 2:**
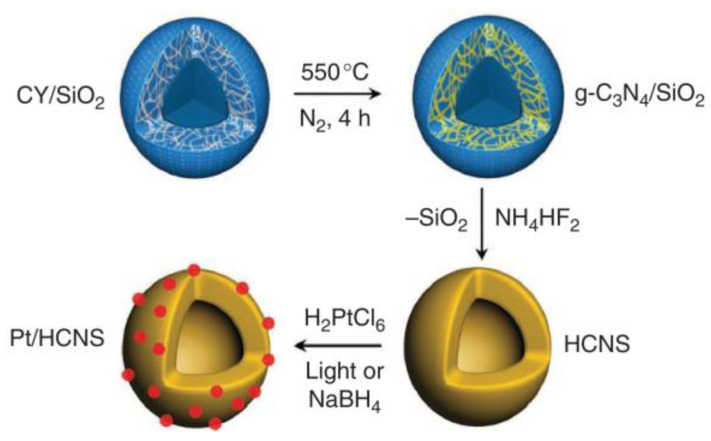
Illustration of HCNS and metal/HCNS composite syntheses. Reprinted with permission from Ref. [34]. Copyright 2012 Nature Publishing Group.

To improve the photocatalytic performance of g-C_3_N_4_, templating strategy has also been applied to synthesize materials with unique appearance, structure, and properties. Due to the high specific surface area and low surface reflection, silica-based materials, including silica spheres, mesoporous silica, and SBA-15, usually serve as hard templates to fabricate g-C_3_N_4_. Sun et al. [34] used silica as a template to prepare a highly stable, hollow g-C_3_N_4_ nanosphere (HCNS) (Figure 2). Adjustable shell thickness performed as a light-harvesting platform for H_2_ evolution under visible light irradiation. However, corrosive reagents (e.g., NaOH, NH_4_HF_2_) are often used to remove silica templates, which are not friendly to the environment. On the contrary, the soft templating method uses ionic liquid as a template or co-template to fabricate g-C_3_N_4_, which is an environmentally friendly and one-step approach to prepare g-C_3_N_4_ with excellent performance. Zhao et al. [35] utilized cyanuric acid-melamine complex and an ionic liquid as soft templates to prepare hollow g-C_3_N_4_ spheres with a specific surface area as high as 84 m^2^ g^−1^. The morphology of g-C_3_N_4_ could be well controlled by adjusting ionic liquid and solvent. It has been demonstrated that the as-prepared hollow mesoporous carbon nitride exhibits ~30 times higher than traditional g-C_3_N_4_ in hydrogen production. Although significant progress has been achieved in the preparation and modification of g-C_3_N_4_, some preparation methods are neither environmentally friendly nor time-saving. Thus, it is necessary to develop green and facile synthesis routes.

## 3. Modification of g-C_3_N_4_

### 3.1. Morphology Control

**Figure 3:**
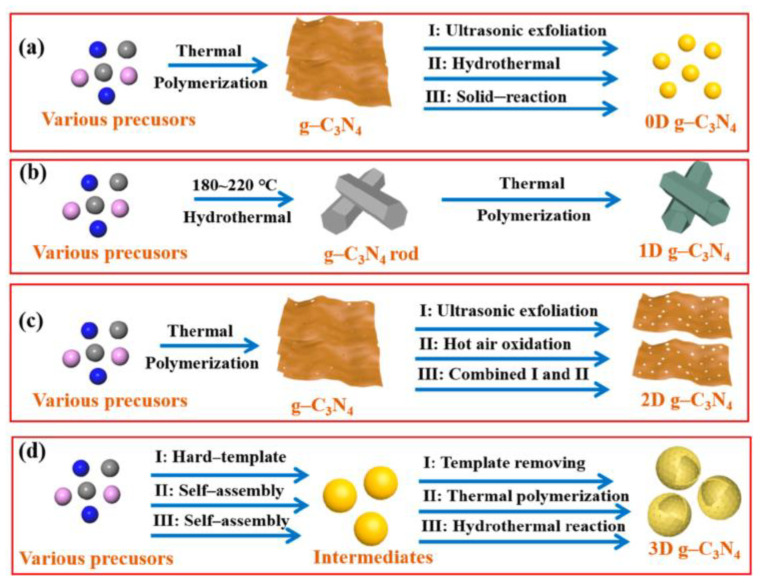
Schematic illustration of different techniques for the synthesis of (**a**) 0D g-C_3_N_4_, (**b**) 1D g-C_3_N_4_, (**c**) 2D g-C_3_N_4_, and (**d**) 3D g-C_3_N_4_. Reprinted with permission from Ref. [25]. Copyright 2021 American Chemical Society.

Morphology control of g-C_3_N_4_ is one of the practical and effective methods to improve its photocatalytic performance. g-C_3_N_4_ can be regulated to different dimensions (Figure 3). Then, with g-C_3_N_4_ having a larger specific surface area, more adequate active sites can be obtained. Moreover, the visible-light response range of g-C_3_N_4_ will be expanded, and the carrier diffusion path will be shortened. This section will discuss 0–3D g-C_3_N_4_-based materials and their catalytic performance.

#### 3.1.1. 0D g-C_3_N_4_

g-C_3_N_4_ dots are new members of the g-C_3_N_4_ family, smaller than 10 nm in size, and have quantum size effect, surface effect, and quantum confinement effect [36]. As the size effect causes the reverse motion of CB and VB, g-C_3_N_4_ with broad absorption (from ultraviolet to visible light) can be obtained. The photogenerated charge carriers favorably migrate to the particle surface for initiating oxidation or reduction reactions. In recent years, a variety of low-cost and size-controlled methods have been developed to prepare g-C_3_N_4_ dots with different physicochemical properties, including hydrothermal treatment [37], ultrasonic exfoliation [38,39], microwave-assisted solvothermal process [40], and solid reaction approach [1]. A hydrothermal and hot-air assisted chemical oxidation method was proposed to prepare g-C_3_N_4_ QDs by etching bulk g-C_3_N_4_ to graphene-like nanosheets [41]. Concentrated H_2_SO_4_ and HNO_3_ etched the nanosheets to produce g-C_3_N_4_ nanoribbons with sizes below 10 nm. Then, 5–9 nm g-C_3_N_4_ QDs can be obtained after the hydrothermal treatment of nanoribbons at 200 °C (pH = 5). The obtained g-C_3_N_4_ QDs exhibited a strong blue emission and upconversion behavior, promising visible-light-driven metal-free photocatalytic systems. Liu et al. [38] synthesized g-C_3_N_4_ QDs by recrystallization and ultrasonic exfoliation from the precursor of dicyandiamide. g-C_3_N_4_ QDs with different sizes (5–200 nm) can be prepared by adjusting ultrasonic time up to 90 min. Chen et al. [40] prepared 0D/2D CNQDs/g-C_3_N_4_ isotype heterojunctions by a simple microwave assisted-polymerization method. The obtained product exhibited excellent photocatalytic performance toward norfloxacin degradation, and its reaction rate was as much as two times higher than pristine g-C_3_N_4_. Zero-dimensional QDs structure materials with nanometer size have a large surface/volume ratio, abundant surface atoms, and unsaturated coordination state, which conduce to their high activity for photocatalysis [42,43]. In addition, the absorption spectra of QDs/g-C_3_N4 will appear blue shift, and band gaps will be broadened, affecting their electronic band structures in photocatalysis [44]. Thus, grafting 0D QDs on g-C_3_N_4_ is a promising approach to creating great reactive active sites and enhancing photoelectric conversion ability to improve photocatalytic activity.

#### 3.1.2. 1D g-C_3_N_4_

**Figure 4:**
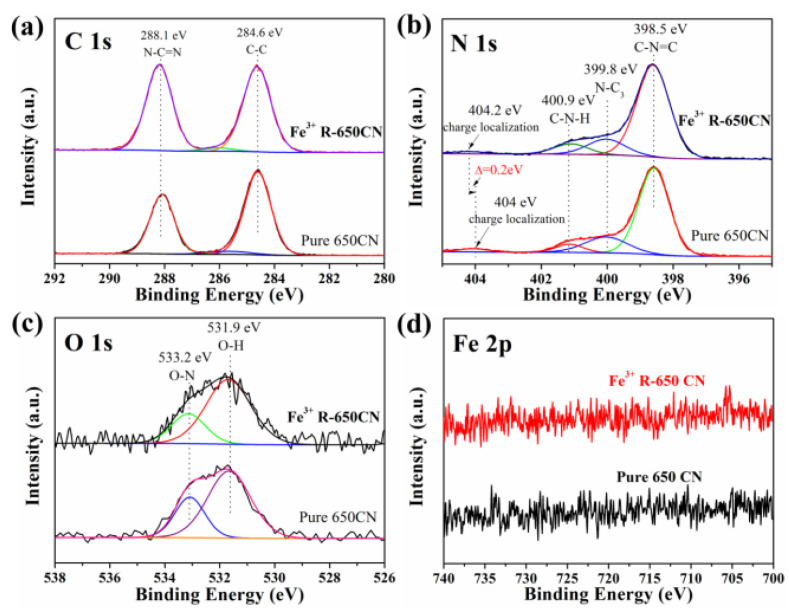
XPS spectra of samples pure 650 CN and Fe^3+^ R-650 CN. (**a**) C 1s. (**b**) N 1s. (**c**) O 1s. (**d**) Fe 2p. Reprinted with permission from Ref. [49]. Copyright 2019 John Wiley and Sons.

As morphology significantly affects materials’ photochemical properties and electron transfer rate, the photocatalytic activity of g-C_3_N_4_ can be improved by adjusting its size and shape. The polymer characteristics of g-C_3_N_4_ make it an excellent flexible structure. One-dimensional g-C_3_N_4_, including nanowires [45], nanorods [46,47], and nanotubes [48], can be prepared by non-metallic hard-template, soft-template, self-template, and template-free methods. Bai et al. [47] found it possible to transform g-C_3_N_4_ from nanoplates to nanorods in a simple reflux way. They compared the photocatalytic activity and intensity of both shapes; results achieved in their study demonstrated that the photocatalytic activity and power of nanorods were ~1.5 and 2.0 times higher than those of nanoplates under visible light, attributable to an increase in active lattice face and elimination of surface defects. Jiang et al. prepared melamine crystals by a transitional metal derived re-crystalline process and then created g-C_3_N_4_ nanotubes with melamine crystals through a thermal polymerizing reaction method. They have applied transitional metal ions (Fe^3+^, Co^2+^, Ni^2+^, and Mn^2+^) in the growing of melamine crystals and have characterized the obtained ion-modified g-C_3_N_4_ nanotubes with XRD, FT-IR spectra, and XPS (Figure 4). It has been demonstrated that Fe^3+^-ion-modified g-C_3_N_4_ nanotubes (Fe^3+^ R-650 CN) exhibited enhanced absorbance at 500 nm and decreased band gap. The hydrogen evolution rate (7538 μmol h^−1^ g^−1^) is almost 13.5-fold than that of conventional g-C_3_N_4_ nanosheets. Mo et al. [48] synthesized defect-engineered g-C_3_N_4_ nanotubes through an efficient self-assembled method, and applied them to hydrogen evolution. Around 6.8% external quantum efficiency was achieved at 420 nm. Among 1D g-C_3_N_4_, nanotubes are superior to others because they allow more effective light absorption, offer more active sites, and invent different electron pathways with tube morphology [25]. Generally, due to the nanometer scale of 1D g-C_3_N_4_-based materials in the radial direction, the diffusion distance (from volume to the surface) of photoexcited charges would be reduced, and the charge separation during photocatalytic reactions would be promoted. In addition, if g-C_3_N_4_-based materials are transformed from 2D to 1D structure, polygonal defects may appear to form the active sites and increase the contact surface of reactions, thus improving the photocatalytic performance.

#### 3.1.3. 2D g-C_3_N_4_

**Figure 5:**
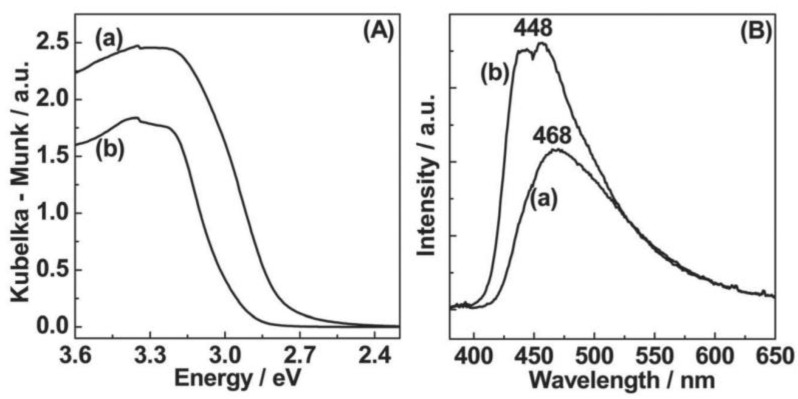
UV-visible absorption spectra (**A**) and fluorescence emission spectra (**B**) of: (a) bulk g-C_3_N_4_ and (b) g-C_3_N_4_ nanosheets. The wavelength of excitation light for fluorescence emission spectra is 350 nm. Reprinted with permission from Ref. [52]. Copyright 2012 John Wiley and Sons.

Bulk g-C_3_N_4_, prepared by the traditional one-step calcination method, has a small specific surface area (10 m^2^ g^−1^), and the photogenerated electrons and holes tend to recombine, which reduces its photocatalytic activity [50]. Since g-C_3_N_4_ has a 2D layered structure connected by van der Waals force, it will produce unique chemical and physical properties as below when it is stripped into multilayer or single-layer nanosheets. First, the large surface area and easy access to active reaction centers facilitate interactions with reactants. Second, nanoscale thickness or even less can minimize the migration distance of charge carriers, ensuring the rapid transport of charge carriers from the bulk phase to the surface of a catalyst. As a result, it would effectively inhibit electron-hole pairs from recombination. Third, the unique two-dimensional flexible planar structure can enhance compatibility with various modification strategies, such as heterojunction construction, cocatalyst modification, and vacancy introduction. This feature further improves the quantum efficiency in a photocatalysis process [51]. Various methods have been developed to prepare g-C_3_N_4_ nanosheets, such as thermal oxidation etching, chemical peel etching, supramolecular self-assembly, and ultrasonic treatment of exudation [1,51]. Among these, thermal oxidation etching is the most common method, which can overcome van der Waals force between layers and peel bulk g-C_3_N_4_ into 2D nanosheets under high-temperature oxidation conditions. Niu et al. [52] prepared g-C_3_N_4_ nanosheets (~2 nm thickness) by thermal oxidation etching of bulk g-C_3_N_4_ in the air. UV-visible absorption (Figure 5A) exhibits a blue shift of the intrinsic absorption edge in the nanosheets. Compared to the bulk (2.77 eV), the nanosheets possess a higher specific surface area and 0.2 eV larger bandgap (Figure 5B). These characteristics benefit electron transport along the in-plane direction and increase the lifetime of photoexcited charge carriers because of the quantum confinement effect. Furthermore, the average hydrogen evolution rate of nanosheets is 170.5 μmol h^−1^ under UV-visible light, which is 5.4 times higher than that of the bulk counterpart. Due to the simplicity of operation, the supramolecular self-assembly method has attracted much attention. For example, conventional melamine-cyanuric acid (MCA) complexes can be obtained by mixing melamine and cyanuric acid in a solution. However, the use of solvent limits the batch preparation of MCA complexes. To address this issue, Liu et al. [53] proposed synthesizing supramolecular precursors through hydrothermal treatment of dicyandiamide and prepared 3D holey g-C_3_N_4_ nanosheets with excellent photocatalytic performance. The precursor exhibited a similar structure to that of conventional MCA. In contrast, their thermal decomposition and morphology were different, which led to the distinction of microstructures, optical properties, charge recombination, photoelectrochemical behavior, and photocatalytic activity. The holey g-C_3_N_4_ can make up for the shortcomings of recombination of charge carriers, retarded visible light utilization, and the limited surface-active sites in the bulk g-C_3_N_4_ catalysts, which could contribute to their outstanding application in photocatalytic hydrogen evolution.

#### 3.1.4. 3D g-C_3_N_4_

**Figure 6:**
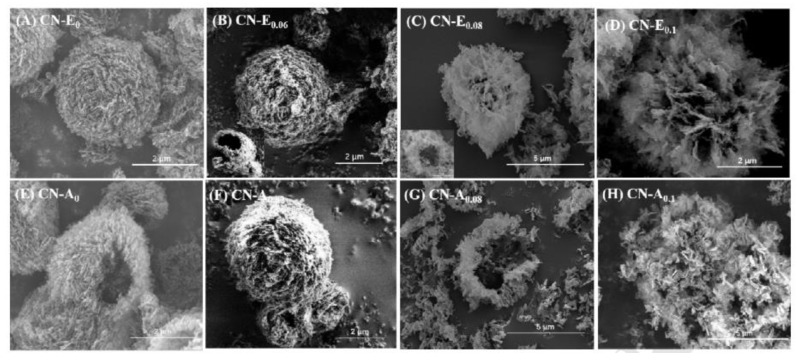
SEM images of hollow mesoporous carbon nitride spheres with different content of ionic liquid. Reprinted with permission from Ref. [35]. Copyright 2018 Elsevier.

Three-dimensional nanostructures are considered an effective way to improve the properties of photocatalytic materials because they can provide a larger specific surface area and more reactive active sites. Although soft and hard template methods have been widely used in the preparation of 3D g-C_3_N_4_, they generally employ toxic substances and organic solvents to remove the template, which limits their application in actual green production. Salt templates have replaced some traditional templates (silica, sodium dodecyl sulfate, anodic alumina) for safe synthesis processes. Qian et al. [54] introduced a simple and effective sodium-chloride-assisted ball milling method to prepare 3D porous g-C_3_N_4_. Three-dimensional cubic sodium chloride particles can be used as an easily removable template to design 3D porous structures and as a limiting structure to prevent aggregation of g-C_3_N_4_ during calcination. The modified 3D interconnecting network structure of g-C_3_N_4_ has a large specific surface area, significantly improving the photocatalytic performance. The hydrogen production rate can be as high as 598 μmol g^−1^ h^−1^ with 3.31% quantum efficiency at 420 nm. In addition, ionic liquids are widely used in many fields due to their excellent fluidity and solubility. In the process of nanomaterial preparation, the ionic liquid can self-assemble into micelles, which impacts the size and morphology of the nanomaterials. For example, Zhao et al. [35] controlled the morphology of hollow mesoporous g-C_3_N_4_ spheres by changing ionic liquid concentration. At a low ionic liquid concentration, the prepared mesoporous g-C_3_N_4_ showed a hollow spherical structure while, at a high ionic liquid concentration, the cyanate-melamine (CM) complex rearranged, which could induce the formation of a flower-like structure with ultrathin nanosheet (Figure 6). Moreover, the hollow as-prepared g-C_3_N_4_ exhibits higher light absorption in the visible range and a faster separation rate of photogenerated hole-electron pairs than bulk C_3_N_4_. In addition, the hydrogen production of as-prepared hollow mesoporous g-C_3_N_4_ exhibits ~30 times higher than traditional g-C_3_N_4_, because of its high surface area.

In addition, some unique g-C_3_N_4_-based structures, like the “seaweed” network, spiral rod, and hollow fusiform, have also been reported [55,56,57,58,59], and a superior photocatalytic activity to bulk samples was observed for higher specific surface areas. Among these morphologies of g-C_3_N_4_, 1D nanoribbons, with a large specific surface area, more active sites, a short diffusion distance, and preferential directions for photo-generated electron-hole carriers, effectively accelerate the catalytic performance of g-C_3_N_4_. Better yet, the 3D morphology assembled from 1D units have depressed agglomeration, increased exposure of active sites, and decreased mass transportation resistance.

### 3.2. Elemental Doping

Heteroatom doping effectively regulates the electrical, optical, and structural properties of semiconductors by introducing active impurities [60]. In general, non-metallic heteroatoms can participate in the C_3_N_4_ lattice and partially replace C or N atoms, while metal atoms can insert into the triangular gap cavity of the g-C_3_N_4_ lattice. Doped metal and non-metal atoms generate intermediate band gaps near CB and VB to regulate the band structure of g-C_3_N_4_, which can effectively realize the separation and transmission of electron-hole pairs and broaden their optical response range [55,60]. Therefore, doping is a prevalent method to improve the photocatalytic performance of semiconductors.

#### 3.2.1. Non-Metal Doping

**Figure 7:**
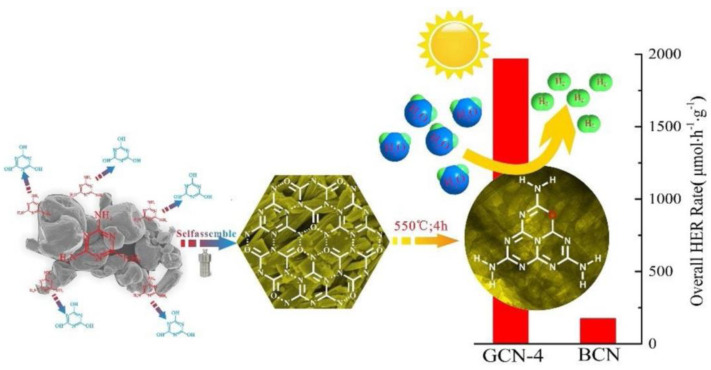
Illustration of the fabrication of porous *O*-doped g-C_3_N_4_ from hydrogen bond-induced supramolecular precursor assembled under hydrothermal treatment. Reprinted with permission from Ref. [64]. Copyright 2018 Elsevier.

Non-metal element doping could maintain the metal-free character of g-C_3_N_4_. Additionally, due to the high ionization energies and electronegativity of non-metals, they can quickly form covalent bonds with other compounds by gaining electrons during the reaction process [61,62]. The introduction of non-metals will break the symmetry of g-C_3_N_4_ and result in a faster separation speed of electron-hole pairs [63]. The ordinary non-metallic doping atoms include O, P, S, B, C, N, and halogens (F, Cl, Br, and I). Among them, the O atom, one of the most typical non-metallic doping elements, has shown extraordinary potential in improving the photocatalytic performance of g-C_3_N_4_. Zhang et al. [64] presented a hydrothermal method and fabricated a porous and oxygen-doped g-C_3_N_4_ photocatalyst for efficient photocatalytic hydrogen production by forming homogeneous supramolecular complexes (Figure 7). They introduced porous structure and heteroatom doping in g-C_3_N_4_ to adjust its active sites and electronic structure for enhanced light harvesting, charge separation, and transfer. Compared with bulk g-C_3_N_4_, the hydrogen evolution activity of the g-C_3_N_4_ photocatalysts is 11.3-fold higher than bulk g-C_3_N_4_. She et al. [65] introduced oxygen in g-C_3_N_4_ and prepared 2D porous ultrathin oxygen-doped g-C_3_N_4_ nanosheets. It has been demonstrated that the band gap was enlarged (~0.20 eV), and the transport ability of photogenerated electrons and the redox ability were improved, which is caused by the quantum confinement effect. Besides, the specific surface area of non-metal doped g-C_3_N_4_ is larger (~20 times) than that of the bulk, which will supply more active sites with adequate quality and offer more adsorption sites. In short, due to the increased bandgap, the introduction of the electrophilic groups and the morphology structure, the electron-hole recombination probability is inhibited and the redox ability will be improved, which contribute to the enhanced photocatalytic activity.

#### 3.2.2. Metal Doping

**Figure 8:**
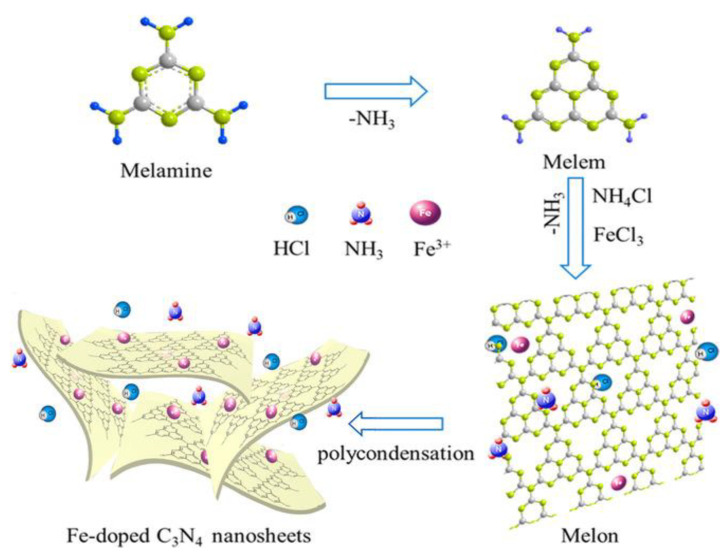
Schematic illustration of the synthesis process of Fe-doped g-C_3_N_4_ nanosheets. Reprinted with permission from Ref. [68]. Copyright 2017 John Wiley and Sons.

In terms of metal-doped g-C_3_N_4_, the porous structure of heptamine and electron-rich sp2 nitrogen atoms can provide sites for metal coordination, and doped metal can easily bind to the three neighboring N atoms in the form of g-C_3_N_4_. Metal element doping, especially alkali, has been demonstrated to reduce the energy gap, supply more active reaction sites, adjust VB position and improve photocarrier separation to enhance the visible light absorption [66]. Due to the uneven distribution of semiconductor charge space, the improvement of photogenerated carrier separation efficiency is increased. In addition, the alkali metal doping can increase the π-conjugated systems and reduce the recombination rate of the semiconductor electron-hole pairs, which will be beneficial to improve the efficiency of H_2_ evolution by photolysis [67]. The ordinary doping metal atoms are K, Na, Ag, Au, Fe, Ni, Pt, etc. Gao et al. [68] designed a simple one-step pyrolysis process to synthesize Fe-doped g-C_3_N_4_ nanosheets with NH_4_Cl as a “dynamic gas template” and FeCl_3_ as a Fe source, respectively (Figure 8). The experimental results show that Fe species may exist at the state of Fe^3+^ and form Fe-N bonds in g-C_3_N_4_, thereby expanding visible light absorption regions and reducing the band gap of g-C_3_N_4_ nanosheets. Moreover, doping specific amounts of Fe could promote exfoliation and increase the specific surface area of g-C_3_N_4_, while excessive Fe might break the sheeting structure. The specific surface area of optimized Fe-doped g-C_3_N_4_ nanosheets reached 236.52 m^2^ g^−1^, which was 2.5 times higher than g-C_3_N_4_ nanosheets. In addition, Deng et al. [69] prepared K^+^ and cyano-group co-doped crystalline polymeric carbon nitride (KC-CCN) by a one-step thermo-polymerization approach. They applied thiourea and potassium thiocyanate as precursors, and the resulting KC-CCN demonstrated a highly crystal structure, stronger light harvesting, and a higher electron-hole separation ratio.

### 3.3. Heterojunction Construction

The key factor that restricts the activity of semiconductor photocatalysts is improving the separation and transport efficiency of electron-hole pairs. Thus, the construction of g-C_3_N_4_-based heterojunction photocatalysts is one of the most common and effective methods. When two semiconductors with different band structures combine to form a heterojunction, effective charge transfer will be included at the interface, improving charge separation and transport efficiency [70,71,72,73,74]. In addition, the light absorption capacity of a photocatalytic system can be enhanced by combining it with narrow-band gap semiconductors. For example, Xu et al. [75] proposed a wet chemical method to fabricate CdS/g-C_3_N_4_ (CSCN) heterojunctions in situ. Through the result of XRD, FTIR, TEM, and optical band gap for CSCN, the formation of heterojunctions was confirmed. The CdS nanoparticles dispersed uniformly on the surface of g-C_3_N_4_ nanosheets and the interfaces between g-C_3_N_4_ and CdS in composites is very close, which can efficiently enhance the electron transfer between the two semiconductors. Comparing the UV–vis DRS spectra of g-C_3_N_4_ (474.9 nm) and CdS (685.7 nm), the absorption thresholds of all CSCN composites locate between that of g-C_3_N_4_ and CdS, which indicates strong visible-light absorption. Among the materials, including the individual CdS, g-C_3_N_4_, and different CSCN composites, CSCN733 possesses the highest adsorption capacity and exhibits the highest methyl orange degradation efficiency, 100% with 40 min adsorption. Liu et al. [76] embedded nanorod-like CoP nanoparticles into g-C_3_N_4_ nanosheets to form CoP-CN heterostructure. The XRD data indicated that the 0.5% CoP-CN hybrid incorporates the representative peaks of g-C_3_N_4_ and CoP with g-C_3_N_4_ demonstrating the main phase. The TEM of 0.5% CoP-CN composites displays a porous and fluffy structure. The binding energy of P 2p_3/2_ is lower than that of P 2p_3/2_, while the binding energy of Co 2p_3/2_ eV is a little higher than that of metallic Co 2p_3/2_. This result indicates that the electron transfer from Co to P to form Co-P covalent bonds results in a small positive charge of Co and negative charge of P. This finding would account for the excellent activity of CoP-based photocatalysts in the HER process, in which Co serves as active center while P performs as the proton acceptor. The flat band potentials of 0.5% CoP-CN were decreased to −0.28 V and the CB tuned upward for more negative potential, achieving more efficient interfacial charge transportation and separation by establishing a certain inner electric field. Furthermore, among the evaluation of photocatalytic water half-splitting for H_2_ production, 0.5% CoP-CN exhibit excellent activity and reached 959.4 μmol h^−1^ g^−1^, which is almost 3.1-fold than that of pristine g-C_3_N_4_ nanosheets. This can be attributed to its decreased over-potentials, more negative photo-reductive potentials, increased interfacial charge transfer efficiency, and higher solar-to-hydrogen efficiency. Ma et al. [77] combined TiO_2_ with g-C_3_N_4_ to form a *Z*-type heterojunction, which can effectively separate photogenerated electrons and holes and improve the photocatalytic activity. However, the utilization of visible light of TiO_2_ is very low because of its large band gap. Thus, to improve this weakness, the Eg of TiO_2_ should be reduced, followed by its potential change of valence band and the conduction band. Such a move would change the heterojunction type, which is not consistent with improving the catalytic efficiency of composite materials to visible light. Therefore, various kinds of g-C_3_N_4_-based heterojunction photocatalysts have emerged to enhance the efficiency of photogenerated carrier separation further and light absorption capacity, including ternary [78,79,80,81,82,83] and other type II [84,85,86,87,88] and *S*-type heterojunction [89,90], Schottky junction [91,92], and van der Waals heterojunction [93,94].

The *S*-type heterojunction is composed of reduction photocatalysts (RPs) and oxidation photocatalysts (OPs) with staggered band structures, similar to the type-II heterojunction but with an entirely different charge-transfer route [95]. *S*-type photocatalysis is an effective way to control charge separation in various photocatalytic reactions. Since Yu et al. first proposed the concept of *S*-type heterojunction in 2019 [96], a large number of studies on the g-C_3_N_4_-based *S*-type heterojunction have been reported, such as Sb_2_WO_6_/g-C_3_N_4_ [97], *S*-doped g-C_3_N_4_/TiO_2_ [98], ZnFe_2_O_4_/g-C_3_N_4_ [99], g-C_3_N_4_/Zn_0.2_Cd_0.8_S-DETA [100], g-C_3_N_4_/Bi/BiVO_4_ [101], WO_3_/g-C_3_N_4_ [102], etc. The Schottky junction photocatalyst has also attracted much attention. Generally, conductor-semiconductor heterojunction has two main combined modes: Schottky junction and ohmic contact. Conductors and semiconductors with different Fermi levels will generate Schottky effects at their contact interfaces to induce internal electric fields to drive charge flow until the system reaches equilibrium. The proper orientation of built-in electric fields will promote the directional separation of charge carriers, leading to the practical generation of photogenerated charge carriers and improving the photocatalytic activity. A large number of g-C_3_N_4_ Schottky junction photocatalysts have been reported, such as CoP/g-C_3_N_4_ [103], CuS/g-C_3_N_4_ [104], Ti_3_C_2_/g-C_3_N_4_ [105], carbon/g-C_3_N_4_ [106], MoO_2_/g-C_3_N_4_ [107], Cu-NPs/g-C_3_N_4_ [108], etc. Recently, van der Waals (vdW) heterojunction has been proposed to regulate the electrical and optical properties of 2D materials accurately. vdW heterojunction not only overcomes the lattice matching limitation for enhancing interfacial charge separation and transfer but also leads to strong electronic coupling between layers to improve catalytic activity [109]. The research on g-C_3_N_4_-based vdW heterojunctions is currently enjoying a boom, and different types have been reported, including phosphorene/g-C_3_N_4_ [110], g-C_3_N_4_/Zn-Ti LDH [111], g-C_3_N_4_/C-doped BN [112], g-C_3_N_4_/COF package-TD [113], etc.

Moreover, metal-organic framework (MOF) materials have exhibited excellent photocatalytic performances due to their unique porous structures and favorable transfer of e^−^ and h^+^ [114,115,116]. However, most MOFs have low stability and weak light response. Therefore, the heterojunction of MOF and g-C_3_N_4_ materials has become popular in recent years. For example, Zhang et al. [117] synthesized a novel hybrid of Zr-based metal-organic framework with g-C_3_N_4_ (UiO-66/g-C_3_N_4_) nanosheets (10:10) by annealing their mixture. The photoelectron can transfer efficiently from the CB of g-C_3_N_4_ to that of UiO-66 through the inner electric field generated by the heterojunction, which is beneficial to decrease the recombination of electron/hole. Together with their porous structures, much more organic dye molecules can absorb on the surface of the heterojunction catalyst, thus facilitating the electron/hole transfer and enhanced photocatalytic activity. Han et al. [114] prepared TPVT (tridentate ligand 2,4,6-tris(2-(pyridin-4-yl)vinyl)-1,3,5-triazine)-MOFs and combined them with g-C_3_N_4_. It has been demonstrated that the TPVT-MOFs@g-C_3_N_4_-10 can reach 56.4 μmol·g^−1^·h^−1^ in CO_2_ reduction, which is 3.2-fold higher than that of g-C_3_N_4_. All these researches have provided a new insight into the design of g-C_3_N_4_ based photocatalysts to deal with the organic dyes in environment.

### 3.4. Modification with Carbon Nanocomposites

**Figure 9:**
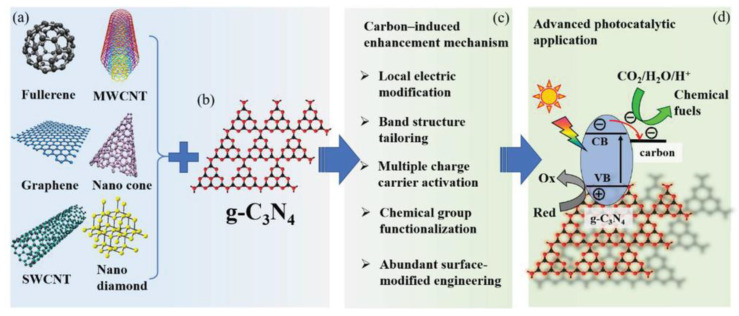
Schematic illustration of enhancement mechanism over carbon-induced g-C_3_N_4_ nanocomposites in photocatalytic action. (**a**) Different types of carbon materials. (**b**) Chemical structure of g-C_3_N_4_; black and red dots represent C and N, respectively. (**c**) The enhanced photocatalytic mechanisms and (**d**) energetic photocatalytic application of carbon-induced metal-free g-C_3_N_4_ nanocomposites. Reprinted with permission from Ref. [121]. Copyright 2020 John Wiley and Sons.

The weak van der Waals forces between layers and the abundant hydrogen bonds in the molecular structure make g-C_3_N_4_ exhibit slow charge transfer kinetics and poor electrical conductivity. Carbon materials have been widely used in photocatalysis due to their low price, good conductivity, high stability, non-toxicity, and harmlessness. Many composite photocatalysts with excellent photocatalytic performance have been prepared by combining carbon nanomaterials (such as fullerene [118], graphene [119], carbon nanotubes [120], etc.) with g-C_3_N_4_, which have unique nanostructures and excellent electron-optical properties (Figure 9). The introduction of carbon material reduces the electron-hole pair recombination rate of photocatalysts and improves the photo absorption, thus improving the photocatalytic performance of g-C_3_N_4_-based materials. g-C_3_N_4_ photocatalysts modified by carbon materials can promote photocatalytic reaction through heterojunction interaction, cocatalyst effect, surface recombination, local charge modification, and other ways [121]. For example, Yuan et al. [122] prepared a graphene-g-C_3_N_4_ composite photocatalyst by calcining graphene with melamine, and excellent photocatalytic degradation performance toward RhB was observed under acidic conditions. Ge et al. [123] prepared multi-walled carbon nanotubes (MWNTs)/g-C_3_N_4_ composite photocatalyst by heating MWNTs and g-C_3_N_4_, in which MWNTs favored the efficient separation of photo-generated charge carriers. As a result, this material exhibited unique performance in photocatalytic H_2_ production under visible light conditions.

**Figure 10:**
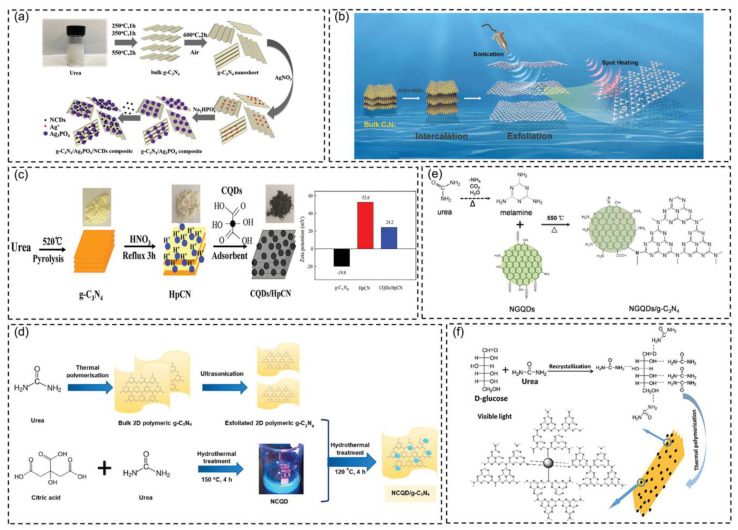
Schematic illustration of the preparation process for g-C_3_N_4_/CD-based nanocomposites through different methods. (**a**) Mechanical mixing method. (**b**) Ultrasonication method. (**c**) Electrostatic attraction method. (**d**) Hydrothermal/solvothermal method. (**e**,**f**) Calcine method. Reprinted with permission from Ref. [130]. Copyright 2021 John Wiley and Sons.

As a new carbon-based nanomaterial, carbon dots (CDs) exhibit excellent up-conversion photoluminescence and remarkable photogenerated charge-carrier transfer and reservoir [124,125,126]. They can also modify g-C_3_N_4_ and broaden optical absorption by reducing the electron-hole pair recombination rate [127,128]. Such features have caused extensive attention to CDs-modified g-C_3_N_4_. For instance, Fang et al. [129] prepared a CDs-modified g-C_3_N_4_ hybrid by dicyandiamide and CDs obtained from the combustion soot of alcohol. Based on the investigation of CDs modification on the structure and photocatalytic activity of g-C_3_N_4_, they found that CDs modification caused the lattice distortion of g-C_3_N_4_, and CDs performed as an electron sink, which could prevent the recombination of photo-generated electron-hole pairs. Ai et al. [130] reviewed the combination methods of g-C_3_N_4_ and CDs (Figure 10) for enhancing photocatalytic performance and indicated that g-C_3_N_4_/CDs hybridization has strong practicability in efficient photocatalytic hydrogen generation, photocatalytic carbon dioxide reduction, and organic pollutant degradation. However, it is still in the early stage. Much effort should be made to develop green and facile synthesis routes and solve the insufficient utilization of visible and near-infrared light.

## 4. Photocatalytic Application in Energy and Environmental Sustainability

The energy and environmental crises have been an ongoing challenge, which is related to the vital interests of people around the globe. How to solve this problem through sustainable development strategies is considered deeply by scientific researchers. Photocatalysis provides a powerful technique for fully utilizing solar in the field of energy conversion [28,131]. Here, we will mainly introduce the photocatalytic application of g-C_3_N_4_ in energy and environmental remediation, including H_2_ production, CO_2_ photoreduction, and pollutant degradation.

### 4.1. H_2_ Production

**Figure 11:**
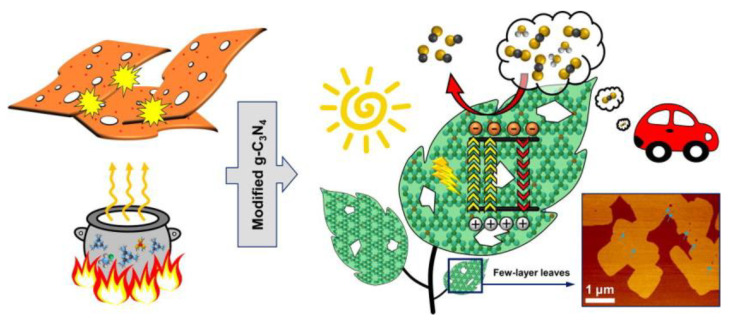
Scheme for a one-pot thermal preparation of amino-functionalized ultrathin nanoporous B-doped g-C_3_N_4_. Reprinted with permission from Ref. [138]. Copyright 2021 Elsevier.

**Figure 12:**
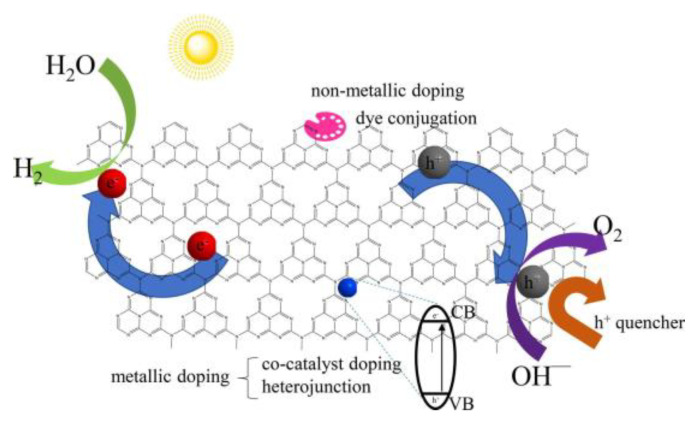
Schematic diagram for the performance comparison on hydrogen evolution between metal- and non-metal-modified g-C_3_N_4_ composites. Reprinted with permission from Ref. [140]. Copyright 2023 Elsevier.

Hydrogen is gathering strong momentum as a pivotal energy transition pillar driven by the global shift toward decarbonization. Nevertheless, 85% of H_2_ is produced from fossil fuel combustion, which generates roughly 500 metric tons of carbon dioxide every year and proffers a challenge and obstacle toward the sustainable living of future generations [132]. Solar-driven photocatalytic H_2_ generation as a promising technology has received extensive attention in addressing the global energy crisis [133,134]. Photocatalytic water splitting for the energy transformation from solar to eco-friendly fuels has been studied for decades with various semiconductor photocatalysts. As a type of semiconductor photocatalyst, g-C_3_N_4_ is simple and inexpensive to fabricate, and has an adequate bandgap (≈2.7 eV) for activation upon sunlight irradiation. Wang’s group first utilized g-C_3_N_4_ in photocatalytic H_2_ evolution [15,135]. Nonetheless, pristine g-C_3_N_4_ is far from satisfactory energy conversion because of its low light energy utilization, low density active sites, and ineffective isolation of the photogenerated excitons. Thus, researchers have proposed numerous strategies to boost the photocatalytic activity of g-C_3_N_4_-based materials for H_2_ production. For example, the g-C_3_N_4_/carbon-dot-based nanocomposites, which possess enormous visible light absorption and applicable energy structures, have been prepared and serve as efficacious photocatalysts in photocatalytic water splitting for H_2_ generation under light illumination [128,134,136,137]. Gao et al. reported hexagonal tubular g-C_3_N_4_/CD-based nanocomposites which exhibited nine times higher than bulk g-C_3_N_4_ in H_2_ production rate [134] and related results indicated that CDs performed as both photosensitizer and electron acceptor. CDs could absorb long wavelength light to extend the visible-light response region and suppress the recombination of electron-hole pairs. Hussien et al. [138] combined four different strategies (non-metal doping, porosity generation, functionalization with amino groups, and thermal oxidation etching) in a one-pot thermal reaction and successfully prepared amino-functionalized ultrathin nanoporous B-doped g-C_3_N_4_ by using NH_4_Cl as a gas bubble template, together with a thermal exfoliation process to produce ultrathin sheets (Figure 11). According to the process, the surface area, adsorption capacity, and charge migration of the as-prepared photocatalyst have been improved, and a 3800 µmol g^−1^ h^−1^ H_2_ generation rate and 10.6% prominent quantum yield were recorded. Li et al. [139] decorated carbon self-doping g-C_3_N_4_ nanosheets with gold-platinum (AuPt) nanocrystals through a photo-deposition route and compared the photocatalytic H_2_ evolution performance of Pt/CCN, Au/CCN, Au/Pt/CCN, and Pt/Au/CCN, in which AuPt/CCN stood out and gave the highest H_2_ generation rate (1135 μmol h^−1^). The excellent performance can be ascribed to the non-plasmon-related synergistic effect of Au and Pt atoms in AuPt nanocrystals. Sun et al. [140] assessed the arrangements of metal- and non-metal-modified g-C_3_N_4_ composites in hydrogen evolution and found that the contribution of dye conjugation in non-metallic g-C_3_N_4_ composites favored their performance (Figure 12). However, the co-catalyst doping strategy was recommended for metallic g-C_3_N_4_ composites. In addition, the hybrid of MOF materials and g-C_3_N_4_ is also a good approach to develop novel photocatalysts. For example, Devarayapalli et al. [141] reported a g-C_3_N_4_/ZIF-67 nanocomposite and obtained a 2084 μmol g^−1^ H_2_ production, which is 3.84-fold greater than that of bare g-C_3_N_4_.

Based on the descriptions mentioned above, Table 1 compares the performance of different g-C_3_N_4_-based materials for photocatalytic H_2_ generation reported within the last three years.

### 4.2. CO_2_ Photoreduction over g-C_3_N_4_

**Figure 13:**
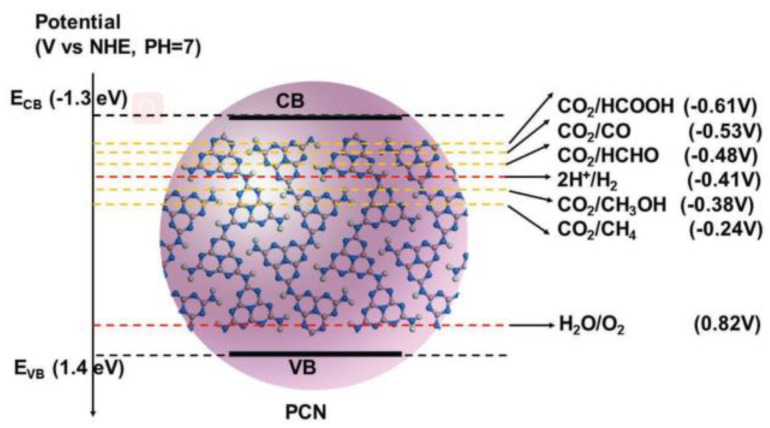
Schematic illustration of energy levels of PCN for photocatalytic CO_2_ reduction. Reprinted with permission from Ref. [177]. Copyright 2019 John Wiley and Sons.

Rising atmospheric levels of CO_2_ and the consumption of fossil fuels raise a concern about the continued reliance on the utilization of fossil fuels for both energy and chemical production [173]. Photocatalytic reduction of CO_2_ is a promising strategy to meet increasing energy needs and reduce the greenhouse effect [174]. Through photocatalytic reduction, CO_2_ can be converted to light oxygenates and hydrocarbons. Photocatalytic CO_2_ reduction is a multielectron transfer process. Fu et al. [175] have listed the possible reaction and corresponding redox potentials and stated that CO_2_ was complicated to reduce at room temperature due to its stable chemical structure. For the complex reaction, five factors, comprising the matching of band energy, separation of charge carrier, kinetic of e- and hole transfer to CO_2_ and reductant, the basicity of photocatalyst, and the strength and coverage of CO_2_ adsorption, are considered to be crucial [176]. As a hot member of photocatalysts, g-C_3_N_4_ has been applied to CO_2_ photo-reduction in recent years because the CB of g-C_3_N_4_ is sufficient to reduce CO_2_ to various hydrocarbons, such as CH_3_OH, CH_4_, HCHO, and HCOOH, etc. (Figure 13) [177].

However, metal-free g-C_3_N_4_ is limited for CO_2_ reduction activity due to its poor ability to activate the C-O bond of CO_2_. To improve the photocatalytic movement of CO_2_ conversion, different metal units have been composited with g-C_3_N_4_ for broadening the absorption response range, and accelerating the charge separation and transfer, such as Pt/g-C_3_N_4_ [178], Co^2+^/g-C_3_N_4_ [179,180], Au/g-C_3_N_4_ [181], etc. Metal nanoparticles acting as cocatalysts could effectively improve the photocatalytic activity and selectivity of CO_2_ reduction. In addition, other methods, including doping, loading cocatalysts and nanocarbons, constructing *Z*-scheme, and heterojunction, have also been employed [16,182,183,184,185,186,187,188,189]. For example, Fu et al. [190] prepared hierarchical porous *O*-doped g-C_3_N_4_ nanotubes (OCN-Tube) through continuing thermal oxidation exfoliation and curling condensation of bulk g-C_3_N_4_. Due to the higher specific surface area, better light harvesting, higher CO_2_ uptake capacity, and superior separation efficiency of photogenerated charge carriers, the OCN-Tube exhibits excellent photocatalytic CO_2_ reduction performance into CH_3_OH. The CH_3_OH evolution rate was as high as 0.88 µmol g^−1^ h^−1^, five times higher than the bulk (0.17 µmol g^−1^ h^−1^). Huo et al. [191] fabricated amine-modified step-scheme (*S*-scheme) porous g-C_3_N_4_/CdSe-diethylenetriamine (A-PCN/CdSe-DETA) by a one-step microwave hydrothermal method. The modification by amine and formation of *S*-scheme heterojunction contributed to the remarkable photocatalytic performance of A-PCN/CdSe-DETA composite in CO_2_ reduction and a CO production rate of 25.87 μmol/(h g) was achieved under visible-light irradiation. Wang et al. [174] reviewed different modification methods of g-C_3_N_4_-based photocatalysts for CO_2_ reduction. They discussed each method (including morphology adjustment, co-catalysts, heterostructures, and doping) and compared the theoretical calculations and experimental results. By morphology adjustment, g-C_3_N_4_ with various shapes can be fabricated, such as rods, tubes, nanosheets, hollow spheres, and honeycomb-like structures. Due to the advantage of cocatalysts (e.g., Au, Ag, Pt, Pd, MXene, AuCu alloy, Pd-Ag), g-C_3_N_4_ with co-catalysts can be widely applied to activate CO_2_ on the surface. Heterojunction with different types is also an effective method to improve the properties of g-C_3_N_4_-based materials. In addition, elemental doping is considered a common method to enhance photocatalytic quantum efficiency by changing the energy band, surface electronic property, and electrical conductivity. Table 2 compares the performance of different g-C_3_N_4_-based materials for photocatalytic CO_2_ reduction reported within the last three years.

### 4.3. Degradation of Organic Pollutants

**Figure 14:**
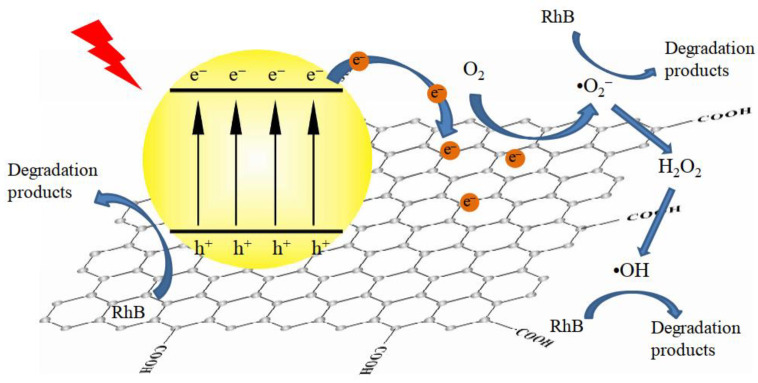
Photocatalytic mechanism of g-C_3_N_4_/rGO (reduced graphene oxide) for the degradation of Rhodamine B. Reprinted with permission from Ref. [122]. Copyright 2015 Elsevier.

Along with rapid population growth and significant industrialization development, large numbers of toxic, hazardous, and endless contaminants invade the environment, threatening to human life, especially a variety of pollutants present in water that are difficult to eliminate or degrade naturally. Photocatalytic degradation of contaminants is a green and efficient technology for coping with sewage [128,223]. Different kinds of g-C_3_N_4_-based materials (Table 3) have been exploited to increase the photodecomposition efficiency of pollutants, such as the constructed heterojunction, loading O_2_-reduction co-catalysts, g-C_3_N_4_/CDs-based nanocomposites, and so on [182,224,225,226]. Generally, under the irradiation of visible light, the photogenerated electrons (e^−^) on the g-C_3_N_4_ catalyst will be excited from VB to CB, leaving holes (h^+^) in the VB. The holes can oxidize pollutants directly or react with H_2_O/OH^−^ to form hydroxyl radicals [227]. When the REDOX potential of g-C_3_N_4_ composites is more negative than O_2_/O_2_^−^, the photogenerated electrons in the material can react with O_2_ to produce O_2_^−^ with strong oxidation capacity [228]. In addition, the resulting O_2_^−^ could be protonated to produce OH [229]. Finally, the RhB dye is degraded to CO_2_ and H_2_O under the action of these free radicals (Figure 14). Chen et al. [230] fabricated a BiFeO_3_/g-C_3_N_4_ heterostructure through mixing-calcining and compared its performance with BiFeO_3_. Around 30% higher photocatalytic efficiency toward RhB dye was observed for the BiFeO_3_/10% g-C_3_N_4_ heterostructure, which was assigned to the contribution of a higher concentration of O_2_^−^. Zhang et al. [231] studied the selective reduction of molecular oxygen on g-C_3_N_4_ and probed its effect on the photocatalytic phenol degradation process. Compared with bulk g-C_3_N_4_, the exfoliated nanosheet yielded a three times improvement in photocatalytic phenol degradation. It has been demonstrated that bulk g-C_3_N_4_ prefers to reduce O_2_ to O_2_^−^via one-electron reduction. At the same time, the photoexcited g-C_3_N_4_ nanosheet facilitates the two-electron reduction of O_2_ to yield H_2_O_2_ because of the formation of 1,4-endoperoxide species. The two-electron reduction of O_2_ on the nanosheet surface boosts hole generation and thus accelerates phenol oxidation degradation [231,232]. Thus, to improve the photocatalytic performance of g-C_3_N_4_, more effort should be devoted to strengthening the solid O_2_-reduction reactions. For example, Liu et al. [83] reported a heterojunction material of K-doped g-C_3_N4 nanosheet -CdS and degraded tetracycline with 94% degradation under visible light in 30 min. In addition, due to the electronegativities, ionic radius differences, and impurity states, element doping is also an effective method to manipulate the electronic structure and physicochemical performance of g-C_3_N_4_-based materials. Gao et al. [68] synthesized Fe-doped g-C_3_N_4_ nanosheets and obtained 1.4- and 1.7-fold higher degradation rates of MB than that of pure g-C_3_N_4_ nanosheets and bulk g-C_3_N_4_, which indicated that the exploitation of efficient g-C_3_N_4_-based photocatalysts with high stabilization and degradation under visible light irradiation would significantly contribute to sewage disposal. Zhang et al. [117] synthesized a novel hybrid of Zr-based metal-organic framework with g-C_3_N_4_ (UiO-66/g-C_3_N_4_) nanosheets and applied a photodegradation of methylene blue, by which a 100% photodegradation was achieved within 4 h under visible light. This research has provided a new insight into the design of g-C_3_N_4_-based photocatalysts to deal with organic dyes in the environment.

## 5. Conclusions and Future Perspective

g-C_3_N_4_-based materials are still a research hotspot in photocatalysis, especially their application in energy and environmental sustainability. Although significant progress has been achieved in the preparation and modification of g-C_3_N_4_, several issues remain to be resolved in future research: (1) Some preparation methods are neither environmentally friendly nor time-saving. Thus, it is necessary to develop green and facile synthesis routes. For example, it should be encouraged to use plant leaves, natural halloysite, and some natural raw materials in the preparation of g-C_3_N_4_-based materials. (2) The absorption ability of available g-C_3_N_4_-based materials to visible and near-infrared light is still low, which is not beneficial to improving solar energy utilization. Coupling g-C_3_N_4_ with visible and near-infrared CDs might be an effective strategy. It would efficaciously improve the e^−^/h^+^ pair separation capability and visible light harnessing capability, thus enhancing the related photocatalytic performance. (3) Some structures of modified g-C_3_N_4_-base materials are complex, and the corresponding photocatalytic reaction mechanisms is not clear yet. Introducing density functional theory could provide insights into the photocatalytic mechanisms via disclosing the materials’ structural, electronic, optical, and other properties. Detailed reaction processes can be performed by using in situ monitoring techniques (e.g., in situ infrared spectroscopy and mass spectrometry) to capture the reactive intermediates. (4) Although microscopic techniques and time-resolved spectroscopy have achieved the study of the steady-state charge distribution and charge transfer dynamics of photocatalysts, tracking the spatiotemporally evolving charge transfer processes in single photocatalyst particles and elaborating their exact mechanism is still a great challenge. Thus, it is significant to develop techniques to map holistic charge transfer processes at the single-particle level, identify where charges go and reveal how long they live on different sites. (5) Finally, the integration of artificial intelligence (AI) and other interdisciplinary techniques will play a tremendous driving role in precisely designing g-C_3_N_4_-based photocatalysts with excellent performance. For example, AI models could be developed to correlate photocatalytic performance with experimental conditions, which may help predict the photocatalytic performance of g-C_3_N_4_-based materials, improve the trial-and-error paradigm, and design new composite structures.

## Figures and Tables

**Table 1:** Photocatalytic H_2_ generation over g-C_3_N_4_-based materials.

Entry	Photocatalyst	Experimental Details	H_2_ Evolution Rate	Reference Material /μmol·g^−1^·h^−1^	Enhancement Relative to Conventional g-C_3_N_4_	Apparent Quantum Efficiency/%	Ref.
1	Cyano-group-modified crystalline g-C_3_N_4_ (CCN0.1)	50 mg CCN, 80 mL lactic acid (10 vol%), 1 wt% Pt/CCN	758.8 μmol·h^−1^	Bulk g-C_3_N_4_ 379.4 μmol·h^−1^	2	1.17%	[142]
2	Ba_5_Nb_4_O_15_/g-C_3_N_4_ (1:20)	420 nm LEDs (3 W single), Pt co-catalyst, 0.05 g, 100 mL oxalic acid	2.67 μmol·h^−1^	g-C_3_N_4_ 1.14 μmol·h^−1^	2.35	6.1	[143]
3	P-doped g-C_3_N_4_ with aromatic ring (AS/P-CN)	300 W Xe, 10 vol% TEOA, 2 wt% Pt, 40 mg	550 μmol·h^−1^·g^−1^	Pristine CN 120 μmol·h^−1^·g^−1^	4.58	0.33	[144]
4	Ni_3_S_2_-NiS_2_/CN-3	300 W Xe, 20 mg Ni_3_S_2_-NiS_2_/CN-X, 90 mL water, 10 mL TEOA	1206.6 μmol·h^−1^·g^−1^	Pure g-C_3_N_4_ 4.01 μmol·h^−1^·g^−1^	300.7		[145]
5	2% MoS_2_-g-C_3_N_4_/Ni_2_P	300 W Xe (>420 nm), 5 °C, 50 mg, 90 mL water, 10 mL of TEOA	298.1 μmol·h^−1^·g^−1^	Pure g-C_3_N_4_ 4.32 μmol·h^−1^·g^−1^	69	2.51% (λ = 420 nm)	[146]
6	HBTiO_2_/g-C3N4 QDs	300 W Xe, 0.025 g, 50 mL 0.25 M Na_2_S, and 0.35M Na_2_SO_3_	10.57 mmol h^−1^·g^−1^	g-C_3_N_4_ 0.32 mmol. h^−1^·g^−1^	33	18.6% 420 nm	[147]
7	0.8 wt.% g-C_3_N_4_/BiVO_4_	200 mL lake water, 500 W halogen, 0.5 M Na_2_SO_4_	21.4 mmol h^−1^			4.27% at 420 nm	[148]
8	g-C_3_N_4_/CoP-4%	350 W Xe, 10 mg, 70 mL water, 10 mL TEOA	936 μmol g^−1^ h^−1^	g-C_3_N_4_—4 wt% Pt 665 μmol g^−1^ h^−1^	1.41		[149]
9	NiCoP-3/C_3_N_4_	300 W Xe (300–780 nm), 100 mg, 10 mL methanol, 90 mL water	159 μmol g^−1^ h^−1^	CoP-3/C_3_N_4_: 63.6 μmol g^−1^ h^−1^ Ni_2_P-3/C_3_N_4_: 4.54 μmol g^−1^ h^−1^	CoP-3/C_3_N_4_: 2.5 Ni_2_P-3/C_3_N_4_: 35	4.2%	[150]
10	15% FeSe_2_/CNNS 2D/2D composite	0.15/0.35 mol/L Na_2_S/Na_2_SO_3_, 30 mg, 300 W Xe	1655.6 μmol g^−1^ h^−1^	C_3_N_4_: 624.8 μmol g^−1^ h^−1^ FeSe_2_: 957 μmol g^−1^ h^−1^	Pristine g-C_3_N_4_: 2.65 FeSe_2_: 1.73		[151]
11	5%-NiCo_2_O_4_/g-C_3_N_4_	300 W Xe, 50 mg, 100 mL solution (10 vol% of TEOA, 3% of H_2_PtCl_6_)	1041.9 μmol g^−1^ h^−1^	g-C_3_N_4_ 521.4 μmol g^−1^ h^−1^	2		[152]
12	CoS/g-C_3_N_4_/NiS ternary photocatalyst	300 W Xe, 100 mg, 85 mL water, 15 mL TEOA	1.93 mmol h^−1^·g^−1^	Bare g-C_3_N_4_ 0.15 mmol h^−1^·g^−1^	12.8	16.4% at 420 nm	[153]
13	5ZnO/ g-C_3_N_4_	0.2 g, 80 mL deionized water, 20 mL methanol	70 µmol h^−1^	g-C_3_N_4_ 8 µmol h^−1^	8.75		[154]
14	20 wt% CuFe_2_O_4_/g-C_3_N_4_	200 W Hg−Xe, 20 mg, Na_2_S/Na_2_SO_3_, TEOA	700.34 μmol g^−1^ h^−1^	g-C_3_N_4_ nanosheets 280.1 μmol g^−1^ h^−1^	2.5	25.09%	[155]
15	Boron-doped g-C_3_N_4_	150 W Xe, 20 mg, 10 vol% TEOA, Pt (1 wt%)	18.2 µmol h^−1^	g-C_3_N_4_ 6.1 µmol h^−1^	3		[156]
16	3.0% β-Bi_2_O_3_/g-C_3_N_4_	500 W Xe, 50 mg, 200 mL glycerol (10% vol.)	8600 µmol g^−1^	Bare β-Bi_2_O_3_ and g-C_3_N_4_ counterparts	>20		[157]
17	Pt/CN-A150 composite	300 W Xe, 10 mg, 100 mL DI water containing 20 vol% TEA	1150.8 µmol h^−1^	Pt/CN-PR 18.2 µmol h^−1^ g-C_3_N_4_	63.2 4.6		[158]
18	g-C_3_N_4_@Ni_3_Se_4_ gC_3_N_4_@CoSe_2_	5 W LED, 10 mg, 30 mL 15% *v/v* TEOA	16.4 µmol∙h^−1^ 25.6 µmol∙h^−1^	Pristine g-C_3_N_4_ 1.9 µmol∙h^−1^	8 13		[159]
19	MoS_2_/g-C_3_N_4_	300 W Xe, 5 mg, 40 mL DI water, 10% *v/v* of TEOA	1787 mmol h^−1^ g^−1^	MoS_2_ g-C_3_N_4_	6 40		[160]
20	Carbon vacancies containing g-C_3_N_4_	300 W Xe, 100 mg, 90 mL deionized water, 10 mL TEOA, 3 wt% Pt	450 µmol h^−1^ g^−1^	Pristine g-C_3_N_4_ 225 µmol h^−1^ g^−1^	2		[161]
21	3 wt% La_2_NiO_4_/g-C_3_N_4_	300 W Xe, 10 mg, 80 mL 20 vol% methanol	312.8 µmol h^−1^ g^−1^	La_2_NiO_4_ 5.8 µmol h^−1^ g^−1^ g-C_3_N_4_ 7.1 µmol h^−1^ g^−1^	53.8 43.9	3.7% 420 nm	[162]
22	18% Ag/AgBr/g-C_3_N_4_	300 W Xe, 50 mg, 90 mL deionized water, 10 mL TEOA	1587.6 µmol h^−1^ g^−1^	g-C_3_N_4_ 59.1 µmol h^−1^ g^−1^	26.9		[163]
23	g-C_3_N_4_/N-doped carbon	300 W Xe, 10 mg, 20 mL 10 vol% TEOA, 0.5 wt% Pt	23.0 µmol h^−1^	g-C_3_N_4_/C 5.9 µmol h^−1^	4		[164]
24	Dendritic fibrous nanosilica/g-C_3_N_4_-0.5	300 W Xe, 10 mg, 0.019 M, 80 µL K_2_PtCl_4_, 5 mL TEOA	4662 µmol h^−1^ g^−1^	Pristine g-C_3_N_4_	7		[165]
25	Ag_0.1_Pd_0.9_/2D CNNs	300 W Xe, 100 mg, 3:1 FA/SF (1.0 M, 4 mL)	231.6 mmol h^−1^	Ag_0.1_Pd_0.9_/2D CNNs under no light	1.87	27.8% 400 nm	[166]
26	g-C_3_N_4_/WO_3_/WS_2_	300 W Xe, 20 mg, 100 mL (20 vol%) TEOA	29 µmol h^−1^ g^−1^	g-C_3_N_4_ nanosheets	7.8	8.9% 420 nm	[167]
27	CeO_2_/g-C_3_N_4_ -6	500 W Xe (400 nm), 0.1g, 100 mL 0.35 M Na_2_SO_3_ and 0.25 M Na_2_S	1240.9 µmol h^−1^ g^−1^	Pure CeO_2_	5.2		[168]
28	Nitrogen vacancies-g-C_3_N_4_	3 W 420 nm LED, 0.02 g, 90 mL H_2_O, 10 mL TEOA, 1% H_2_PtCl_6_ 2H_2_O (10 mg/mL)	3259.1 µmol h^−1^ g^−1^	Pristine g-C_3_N_4_	8.7		[169]
29	Black Cu-g-C_3_N_4_ nanosheets composite	300 W Xe, 10 mg 100 mL (1:9 TEOA: Water)	526 µmol h^−1^ g^−1^	g-C_3_N_4_: 280			[170]
30	Amino-group-rich porous g-C_3_N_4_ nanosheets (AP-CN 1.0)	420-nm LED, 0.05 g, 80 mL 10 vol% TEOA, 1 wt% Pt	130.7 µmol h^−1^	Bulk g-C_3_N_4_	4.9	5.58	[171]
31	0.3-MoS_2_/g-C_3_N_4_	300 W Xe, 100 mg, 50 mL, deionized water and 5 mL TEOA	12 mmol h^−1^ g^−1^	Pristine g-C_3_N_4_ g-C_3_N_4_ (Pt)	218 3	0.5% 420 nm	[172]
32	g-C3N4/ZIF-67	MaX 303 solar simulator, 20 mg, 0.5 M Na_2_SO_4_	2084 μmol g^−1^	Bare g-C_3_N_4_ 541 μmol g^−1^	3.84		[141]
33	2D/2D ZnCoMOF/g-C3N4	300 W Xe, 10 mg, 0.1 mL DMF	1040.1		Bulk g-C_3_N_4_: 33.2 2D g-C_3_N_4_: 3.5		[116]
34	PCN-222(M)/g-C_3_N_4_	300 W Xe, 10 mg, 25 mL TEOA	1725.5 µmol h^−1^ g^−1^		PNi: 19.3 CN: 3.7		[115]

**Table 2:** Photocatalytic CO_2_ reduction over g-C_3_N_4_-based materials.

Entry	Photocatalyst	Experimental Details	Productivity /μmol·g^−1^·h^−1^	Reference Material /μmol·g^−1^·h^−1^	Enhancement Relative to Conventional g-C_3_N_4_	Apparent Quantum Efficiency/%	Ref.
1	Ni/g-C_3_N_4_-0.5 catalyst	300 W Xe, 94–95 kPa, 10.0 mg, deionized water	CO: 19.9	g-C_3_N_4_: 4.8	4.1		[192]
2	*S*-scheme CuWO_4_ @ g-C_3_N_4_ core-shell microspheres	300 W Xe (≥420 nm), 0.1 g NaHCO_3_, 0.5 mL 4M HCl	CO: 4.15 CH4: 0.12	g-C_3_N_4_ CO: 1.56 CH_4_: 0.02	2.7		[193]
3	Hydroxyl-modified g-C3N4/flower-like Bi_2_O_2_CO_3_ composites	blue LED (4 × 3 W) 450 ± 20 nm, 40 mg, deionized water	CO: 26.69	Pristine g-C_3_N_4_ CO: 1.47	18.2		[194]
4	*Z*-scheme g-C3N_4_/BiVO_4_ (CN/BVO) heterojunction	300 W Xenon lamp, 0.05 g, 5 mL water	CO: 48	Pristine BVO CO: 2	24		[195]
5	Ultrathin dimension-matched *S*-scheme Bi_3_NbO_7_/g-C_3_N_4_ hetero-structure	Solar simulator, 50 mg, deionized water, 1.3 g Na_2_CO_3_, 2.0 mL H_2_SO_4_	CH_4_: 37.59	Ultrathin g-C_3_N_4_ nanosheets CH_4_: 2.5	15		[196]
6	Van der Waals (vdW) heterojunction composite combining g-C_3_N_4_ with nitrogen vacancies and Tp-Tta COF	300 W Xe, 20 mg, 15 mg bpy, 1 μmol CoCl_2_, acetonitrile, water, TEOA	CO: 11.25	Pristine g-C_3_N_4_ CO: 0.25, g-C_3_N_4_ (NH) CO: 3.5	Pristine g-C_3_N_4_: 45 g-C_3_N_4_ (NH): 3.2		[197]
7	C-NHx-rich 24 g-C_3_N_4_	300 W Xe (420 nm), 10 mg g-C_3_N_4_, 10 mL deionized water, pH at 30 ℃	CO: 185.7	g-C_3_N_4_ CO: 2.5	g-C_3_N_4_: 74		[198]
8	g-C_3_N_4_/3DOM-WO_3_	300 W Xe (≥420), water, 0.1 g catalyst, 2 mL deionized water	CO: 48.7 CH4: 7.5 O2: 44.5	Pure g-C_3_N_4_ nanosheets CO: 25.2 CH_4_: undetected	Pure g-C_3_N_4_ nanosheets CO: 1.9		[199]
9	g-C_3_N_4_/rGO composites	300 W Xe, 3 mg mL^−1^ catalysts, 5 mL 0.2 M NaHCO_3_, illuminated 12 h	CH_3_OH: 114	CdIn_2_S_4_/g-C_3_N_4_ CH_3_OH: 42.7	CdIn_2_S_4_/g-C_3_N_4_: 2.67	0.63	[200]
10	15% LaCoO_3_ loaded g-C_3_N_4_	35 W Xe (420 nm), 50 mg photocatalyst, pressure 0.30 bar	CO: 135.2 CH_4_: 48.5	Pristine La-CoO_3_ CO: 110 CH_4_: 28.5 g-C_3_N_4_ CO: 114 CH_4_: 30.4	Pristine LaCoO_3_ CO: 1.2 CH_4_: 1.7 g-C_3_N_4_ CO: 1.18 CH_4_: 1.59		[201]
11	Bi_2_O_2_(NO_3_)(OH)/g-C_3_N_4_	300 W Xe, 20 mg samples, 3 mL DI water	CO: 14.84	BON CO: 0.94 g-C_3_N_4_ CO: 3.29	pure BON:15 g-C_3_N_4_: 3.5		[202]
12	*Z*-scheme SnS2/gC3N4/C	300 W Xe, 0.05 g catalyst 100 mL deionized water, 25 °C, 5 h	CO: 40.86	Pristine g-C_3_N_4_ CO: 7.42	Pristine g-C_3_N_4_ 5.5		[203]
13	ND/g-C_3_N_4_	300 W Xe (>420 nm), 30 mg catalyst, 18 mL acetonitrile, 6 mL water, 1 μmoL CoCl_2_⋅6H_2_O	CO: 10.98	CO: 0.59	bulk g-C_3_N_4_ 18.6		[204]
14	ZnIn_2_S_4_ nanosheets modified hexagonal g-C_3_N_4_ tubes	300 W Xe (420 nm), 4 mg, 2 mL water, 1 mL of triethanolamine, 3 mL acetonitrile, 15 mg 2′2-bipyridine (bpy) and 2 µmol of CoCl_2_	CO: 883	HCNT: 66 ZIS: 367.9	HCNT: 13 ZIS: 2.4	8.9%	[205]
15	g-C_3_N_4/_covalent triazine framework (CN/CTF 2.5%)	300 W Xe, 5 mg catalyst in 4 mL acetonitrile, 1 mL Co(bpy)_3_Cl_2_ triethanolamine	CO: 151.1	CTF: 5.93 CN: 60.44	CTF: 25.5 CN: 2.5		[206]
16	g-C_3_N_4_-W_18_O_49_ nanocomposite	300 W Xe, 50 mg catalyst in 1 mL deionized water	CH_4_: 1.38	g-C_3_N_4_: 0.17 W_18_O_49_: 0.12	g-C_3_N_4_: 8.12 W_18_O_49_: 11.5		[207]
17	SnS_2_/Au/g-C_3_N_4_ embedded structure	300 W Xe, 20 mg, 100 mL water and TEOA, 140 kPa	CO 93.81 CH_4_ 74.98				[208]
18	Bi_3_O_4_Cl/20%g-C_3_N_4_	300 W Xe, 0.05 g catalyst, 5 mL H_2_O	CO: 6.6 CH_4_: 1.9	Pure g-C_3_N_4_ CO: 2.2, CH_4_: 0.6 Bi_3_O_4_Cl CO: 2.9 CH_4_: 0.7	g-C_3_N_4_ CO: 3 CH_4_: 3.17 Bi_3_O_4_Cl CO: 2.28 CH_4_: 2.71	Bi_3_O_4_Cl/20% g-C_3_N_4_ is 0.14% under 365	[209]
19	2D/2D g-C_3_N_4_/NaBiO_32_H_2_O (10 CN/NBO)	300 W Xe, 25 mg, deionized water, 1.2 g NaHCO_3_, 2 mL H_2_SO_4_ (1:1 vol)	CO: 110.2 CH_4_: 43.8	Pure CN CO: 65.68 CH_4_: 0.42 NBO CO: 26.45 CH_4_: 4.81	Pure CN CO: 1.68 CH_4_: 104.3 NBO CO: 4.16 CH_4_: 9.1		[210]
20	Ultrathin nanosheet g-C_3_N_4_ (NS-g-C_3_N_4_)	300 W Xe (420 nm), 0.1 g photocatalyst, 50 mL 50 g/L KHCO_3_	CO: 38 μmol/L with 6 h	Bulk g-C_3_N_4_ CO: 6.56 μmol/L	CO: 5.8		[211]
21	3% CdS-g-C_3_N_4_ heterostructures	300 W Xe (420 nm), 1 g/L catalyst 100 mL H_2_O, 80 ℃, 125 mg Na_2_CO_3_, 0.25 mL HCl (4 M)	CH_3_OH: 192.7	CdS CH_3_OH: 47.1 pristine g-C_3_N_4_ CH_3_OH: 32.6	CdS: 4.1 pristine g-C_3_N_4_: 5.9		[212]
22	*Z*-scheme ZnO/Au/g-C_3_N_4_ micro-needles film (3-ZAC)	300 W UV–vis lamp, fiberglass sheets, 0.4 M Pa	86.2 μmol m^−2^ h^−1^	Pure ZnO 19.16 μmol m^−2^ h^−1^	Pure ZnO film: 4.5		[213]
23	rGO/R-CeO_2_/g-C_3_N_4_	300 W Xe, 100 mg catalysts, 100 mL 1 M NaOH, 1 mmol TEOA, 0.4 MPa	CO:15.8 CH_4_: 8.15	CO: 3.95 CH_4_: 1.36	Pure g-C_3_N_4_ CO: 4 CH_4_: 6		[214]
24	g-C_3_N_4_/ZnO composites	300 W Xe (λ ≥ 420 nm), 60 mg catalysts, 1.60 g NaHCO_3_, H_2_SO_4_ (40%, 5.0 mL)	CH_4_: 19.8 CO: 0.37	g-C_3_N_4_ CH_4_: 0.9 CO: 4.8	g-C_3_N_4_ CH_4_: 22 CO: 0.078		[215]
25	K-CN-7	300 W Xe, 50 mg catalyst, 200 μL deionized water, 1 cm × 3 cm ITO glass; 0.5 M Na_2_SO_4_	CO: 8.7	Ordinary g-C_3_N_4_ CO: 0.348	Ordinary g-C_3_N_4_: 25		[216]
26	g-C_3_N_4_/CdS heterostructure nanocomposite	150 W Xe, 20 mg catalyst, 7 mL acetone nitrile, 0.5 mL H_2_O, 0.5 g TEOA, 4 µmol [Co(bpy)_3_]Cl	CO: 234.6	CN-12: 58.65 CdS: 9.2	CN-12: 4.0 CdS: 25.5		[217]
27	Porous structure g-C_3_N_4_ with nitrogen defect photocatalysts (DCN-P)	300 W Xe, 0.05 g catalyst, 100 mL deionized water	CO, 19.7 CH_4_: 37.1	Bulk g-C_3_N_4_ CO: 4.1 CH4: 9.6	Bulk gC_3_N_4_ CO: 4.8 CH_4_: 3.86		[218]
28	g-C_3_N_4_/Bi_2_O_2_[BO_2_(OH)] (CNBB-_3_)	300 W Xe, 20 mg sample, 2 mL deionized water, 1.7 g Na2CO3, 15 mL H2SO4	CO: 6.09	Pristine g-C_3_N_4_ CO: 2.19	Pristine g-C_3_N_4_ 2.78		[219]
29	Type-Ⅱ heterojunction of Zn_0.2_Cd_0.8_S/g-C_3_N_4_	300 W Xe, 80 °C, 0.6 MPa, 10 mg catalyst, 20 mL H_2_O	CH_3_OH: 11.5 ± 0.3	Zn_0.2_Cd_0.8_S: CH_3_OH: 4.4 ± 0.2 g-C3N4: CH_3_OH: 4.2 ± 0.1	Zn_0.2_ Cd_0.8_S: 2.6 g-C_3_N_4_: 2.7		[220]
30	3ZIF/1.5Au-PCN	300 W Xe, 0.1 g, 50 mL H_2_O	CO: >10 CH4: >4		Pristine g-C_3_N_4_ 8		[221]
31	TPVT-MOFs@g-C_3_N_4_-10	LED light, 1 mg, 1 mL dichloromethane	CO: 56.4	Pure g-C_3_N_4_: 17.5	Pure g-C_3_N_4_ 3.2		[114]
32	NH2-MIL-101(Fe)/g-C3N4-30 wt%	300 W Xe, 2 mg	CO: 132.8	g-C_3_N_4_: 19.2	g-C_3_N_4_ 6.9		[222]

**Table 3:** Photocatalytic degradation of pollutants over g-C_3_N_4_-based materials reported within the last three years.

Entry	Photocatalyst	Pollutant Concentration	Light Source	Degradation Efficiency/%	Ref.
1	5% g-C_3_N_4_-TiO_2_	Acetaminophen: 0.033 mM	300 W Xe (>400 nm)	99.3 in 30 min	[233]
2	3ZIF/1.5Au-PCN	Bisphenol A	350 W Xe (>420 nm)	>85%	[221]
3	Cu(tmpa)/20%CN	Congo red: 100 mg·L^−1^	150 W Xe	98.2% in 3 min	[234]
4	BiO-Ag(0)/C_3_N_4_@ ZIF-67	Congo red: 12 mg·L^−1^	Natural sunlight	90% in 150 min	[13]
5	C_3_N_4_/RGO/Bi_2_Fe_4_O_9_	Congo red: 10 mg·L^−1^	LED 30 W	87.65% in 60 min	[235]
6	g-C_3_N_4_/Co-MOF	Crystal violet: 4 ppm	MaX 303 solar simulator (50 mW/cm)	95% in 80 min	[141]
7	Honeycomb-like g-C_3_N_4_/CeO_2_-x	Cr (VI): 20 mg·L^−1^	300 W Xe (>420 nm)	98% in 150 min	[236]
8	Sm_6_WO_12_/g-C_3_N_4_	Levofloxacin: 10 mg·L^−1^	150 Mw cm^−2^ tungsten lamp	98% in 70 min	[237]
9	O-g/C_3_N_4_	Lincomycin: 100 mg·L^−1^	PCX50C system (>420 nm)	99% within 3 h	[238]
10	ZnO-modified g-C_3_N_4_	Methylene blue: 10 ppm	200 W tungsten lamp (>420 nm)	97% in 80 min	[239]
11	Wood-like g-C_3_N_4_@WDC	Methylene blue: 20 mg·L^−1^	300 W Xe (>400 nm)	98% in 60 min	[240]
12	BiO-Ag(0)/C_3_N_4_@ ZIF-67	Methylene blue: 12 mg·L^−1^	Natural sunlight	96.5% in 120 min	[13]
13	Cerium-based GO/g-C_3_N_4_/Fe_2_O_3_	Methylene blue: 10 mg·L^−1^	Light bulb	70.61% in 45 min	[14]
14	Ytterbium oxide-based GO/g-C_3_N_4_/Fe_2_O_3_	Methylene blue: 10 mg·L^−1^	Light bulb	83.5% in 45 min	[14]
15	Cu(tmpa)/20%CN	Methylene blue: 10 mg·L^−1^	150W Xe	92.0% within 20 min	[234]
16	C_3_N_4x_/AgO_y_@Co_1-x_Bi_1-y_O_7_	Methylene blue: 25 mL 10 mM	100 W tungsten bulb	96.4% in 120 min	[12]
17	Ternary composites of Zr-MOF combined with g-C3N4 and Ag_3_PO_4_	Methylene blue: 10 mg·L^−1^	85-watt tungsten lamp outdoor/solar light in an open air	95% within 240 93% within 105 min	[241]
18	PSCN/Ag@AgI/WO_3_	Malachite green: 1 × 10 ^−4^ mol dm^−3^	35 W LED	90% in 60 min	[242]
19	Cu(tmpa)/20%CN	Malachite green: 30 mg·L^−1^	150W Xe	92.9% in 35 min	[234]
20	20% g-C_3_N_4_/Bi_4_O_5_I_2_	Methyl orange: 20 mg·L^−1^	350 W Xe	0.164 min^−1^	[243]
21	Cu(tmpa)/20%CN	Methyl violet: 10 mg·L^−1^	150W Xe	92.0% in 60 min	[234]
22	MnCo_2_O_4_/g-C_3_N_4_	Nitrobenzene: 40 mg L^−1^	CMCN2/PMS system	96.7% in 240 min	[244]
23	C_3_N_4x_/AgO_y_@Co_1-x_Bi_1-y_O_7_	Oxytetracycline: 25 mL 25 mM	100 W tungsten bulb	93% in 160 min	[12]
24	g-C_3_N_4_/WO_3_/WS_2_	Rhodamine B: 25 mg L^−1^	300 W Xe (>420 nm)	96.2% in 20 min	[167]
25	Flower-like Bi_12_TiO_20_/g-C_3_N_4_	Rhodamine B: 20 mg·L^−1^	150 mW·cm^−2^ Xe (>420 nm)	100% in 30 min	[245]
26	CdS/CQDs/g-C_3_N_4_	Rhodamine B: 10 mg·L^−1^	300 W Xe (>420 nm)	100% in 20 min	[246]
27	Ytterbium oxide-based GO/g-C_3_N_4_/Fe_2_O_3_	Rhodamine B: 10 mg·L^−1^	Light bulb	67.11% in 45 min	[14]
28	Cerium-based GO/g-C_3_N_4_/Fe_2_O_3_	Rhodamine B: 10 mg·L^−1^	Light bulb	63.08% in 45 min	[14]
29	Fish-scale g-C_3_N_4_/ZnIn_2_S_4_	Tetracycline: 10 mg·L^−1^	300 W Xe (>420 nm)	74% in 30 min	[247]
31	Flower-like Co_3_O_4_/g-C_3_N_4_	Tetracycline: 15 mg·L^−1^	350 W Xe (>420 nm)	85.32% in 120 min	[248]
31	10 wt% CuAl_2_O_4_/g-C_3_N_4_	Tetracycline hydrochloride: 100 mg·L^−1^	300 W Xe (>400 nm)	89.6% in 60 min	[249]
32	CO-C_3_N_4_	Tetracycline hydrochloride: 10 mg·L^−1^	300 W Xe (>420 nm)	97.77% (PMS) in 40 min	[250]
33	ZIF-67/g-C_3_N_4_	Venlafaxine: 10 mg·L^−1^	-	27.75% within 120 min	[251]
34	ZIF-67/MIL-100(Fe)/g-C_3_N_4_	Venlafaxine: 10 mg·L^−1^	-	100% within 120 min	[251]
35	ZIF-67/MOF-74(Ni)/g-C_3_N_4_	Venlafaxine: 10 mg·L^−1^	-	91.8% within 120 min	[251]

## Data Availability

Not applicable.
